# A brief review on DNA vaccines in the era of COVID-19

**DOI:** 10.2217/fvl-2021-0170

**Published:** 2021-11-26

**Authors:** Maryam Shafaati, Massoud Saidijam, Meysam Soleimani, Fereshte Hazrati, Rasoul Mirzaei, Bagher Amirheidari, Hamid Tanzadehpanah, Sajad Karampoor, Sima Kazemi, Bahram Yavari, Hanie Mahaki, Mohsen Safaei, Fatemeh Rahbarizadeh, Pouria Samadi, Yaghoub Ahmadyousefi

**Affiliations:** ^1^Department of Microbiology, Faculty of Sciences, Jahrom Branch, Islamic Azad University, Jahrom, Iran; ^2^Department of Medical Biotechnology, School of Advanced Medical Sciences & Technologies, Hamadan University of Medical Sciences, Hamadan, Iran; ^3^Research Center for Molecular Medicine, School of Medicine, Hamadan University of Medical Sciences, Hamadan, Iran; ^4^Department of Pharmaceutical Biotechnology, School of Pharmacy, Hamadan University of Medical Sciences, Hamadan, Iran; ^5^Department of Microbiology, School of Medicine, Hamadan University of Medical Sciences, Hamadan, Iran; ^6^Department of Pharmaceutical Biotechnology, Faculty of Pharmacy, Kerman University of Medical Sciences, Kerman, Iran; ^7^Extremophile and Productive Microorganisms Research Center, Kerman University of Medical Sciences, Kerman, Iran; ^8^Department of Virology, School of Medicine, Iran University of Medical Sciences, Tehran, Iran; ^9^Vascular & Endovascular Surgery Research Center, Mashhad University of Medical Sciences, Mashhad, Iran; ^10^Department of Medical Biotechnology, School of Advanced Technologies, Shahrekord University of Medical Sciences, Shahrekord, Iran; ^11^Department of Medical Biotechnology, Faculty of Medical Sciences, Tarbiat Modares University, Tehran, Iran

**Keywords:** COVID-19, DNA, DNA vaccines, nucleic acid vaccines, SARS-CoV-2, vaccines

## Abstract

This article provides a brief overview of DNA vaccines. First, the basic DNA vaccine design strategies are described, then specific issues related to the industrial production of DNA vaccines are discussed, including the production and purification of DNA products such as plasmid DNA, minicircle DNA, minimalistic, immunologically defined gene expression (MIDGE) and Doggybone™. The use of adjuvants to enhance the immunogenicity of DNA vaccines is then discussed. In addition, different delivery routes and several physical and chemical methods to increase the efficacy of DNA delivery into cells are explained. Recent preclinical and clinical trials of DNA vaccines for COVID-19 are then summarized. Lastly, the advantages and obstacles of DNA vaccines are discussed.

Recent advances in biotechnology have revolutionized medicine and offered pioneering solutions to unmet clinical needs. Vaccines are one of the most important medical interventions. There are different types of vaccine platforms for infectious diseases and cancer such as live-attenuated, whole-inactivated, subunit, virus-like particles, viral-vector, mRNA and DNA vaccines [1]. Nucleic acid-based diagnostic, prognostic and therapeutic platforms are promising tools that are replacing older protein-based platforms due to their unique properties such as thermostability, resistance to denaturation and simple storage [2]. It has been documented that a good vaccine platform should be rapid, simply developed, reproducible, thermostable and manufactured with reducing development costs and risks [3]. The DNA platform addresses many of these goals. Wolff *et al.* were the first to show that the injection of naked plasmid DNA in the mouse muscle results in a local expression of the transgene [4]. This research was a turning point in the use of DNA in vaccine development.

DNA vaccines are DNA vehicles such as bacterial plasmids, minicircle DNA or linear, covalently-closed minimalistic expression constructs including minimalistic, immunologically defined gene expression (MIDGE) DNA and Doggybone™ DNA that contain at least one eukaryotic expression cassette encoding for the antigen of interest. The expression cassettes usually consist of a eukaryotic promoter/enhancer, the antigen gene and a poly(A) signal sequence, which are essential for the expression of the antigen in eukaryotic cells (e.g., muscular cells) [5–7]. DNA vaccines have shown compelling safety and immunogenicity in preclinical studies [8]. Several DNA vaccines are currently licensed for veterinary use in large animals such as horses as well as small animals such as chickens [9,10]. The results from clinical trials of DNA vaccines for West Nile virus (WNV) [11,12], Ebola and Marburg viruses [13,14] and SARS-CoV-2 [15–19] have shown that antibodies are generated in humans a few weeks after immunization. However, there are also many cases of poor immunogenicity in clinical trials. Target antigen and optimization of construct, formulation and delivery methods appear to be key elements in the immunogenicity of DNA vaccines [8]. This review will discuss the design, production, delivery and administration of DNA vaccines, factors that may improve the immunogenicity of DNA vaccines, and summarize the recent preclinical and clinical trials of DNA vaccines for COVID-19.

## Design of DNA vaccines

The plasmid DNA vaccines are comprised of a bacterial origin of replication and at least one antibiotic resistance gene as a selectable marker. It was shown that bacterial backbone can reduce gene expression in mammalian cells [20]. The formation of heterochromatin in bacterial sequences spreading into the expression cassette may be one of the reasons for the silencing of transgene expression [21]. In addition, it was found that increasing the A/T sequence composition in plasmid antibiotic resistance genes can increase the stable transcription of backbone genes as well as adjacent expression cassettes in mice [22]. Changes in sequence composition and deletion of bacterial backbone sequences in DNA vaccines may increase antigen expression. Minicircle DNA, MIDGE and Doggybone™ are DNA constructs composed of the gene expression cassette(s) without the bacterial backbone of plasmids [5–7].

Sometimes, we need to express multiple genes in a single DNA vaccine, for example, designing multi-antigen DNA vaccines or expressing a genetic adjuvant combined with the antigen. In this regard, three strategies were used: first, we can use different expression cassettes for each gene with individual promoters for independent expression of multiple transgenes; second, we can use bi-cistronic or multi-cistronic vectors with a single promoter for the expression of multiple genes which are separated by internal ribosome entry site (IRES) elements for independent translation of multiple genes; third, we can use a virus-derived T2A sequence instead of IRES between genes, where, after translation, the corresponding peptide sequence is recognized and cleaved by an endogenous protease [23–27].

The genetic material of a DNA vaccine must first enter the nucleus for subsequent transcription of the encoding genes of antigens or genetic adjuvants. Then, the transcribed mRNA(s) are exported from the nucleus into the cytoplasm for translation. The efficient DNA transfer to the cell nucleus is an important barrier for the expression of transgenes in DNA vaccines, especially for mitotically inactive cells such as antigen-presenting cells (APCs) [27]. Certain DNA sequences such as the simian virus (SV) 40 enhancer have a nuclear localization signal (NLS), and binding of specific transcription factors to this NLS signal in the cytoplasm leads to active nuclear transport of DNA [28]. In addition, insertion of some tissue-specific transcription factor-binding sequences in DNA plasmids may lead to tissue-specific nuclear import of plasmid DNA [29]. Alternatively, some DNA binding proteins such as NFκB (p50) and engineered NLS-tetracycline repressor can be used to form protein–DNA complexes before administration, improving the nuclear localization of DNA in cells [30–32]. In addition, covalent or noncovalent conjugation of a virus-derived NLS peptide to either natural or synthetic polycation DNA condensing agents or directly to DNA could be another strategy to improve the nuclear translocation of exogenous DNA [33–37].

Typically, viral promoters such as the human cytomegalovirus (CMV) promoter which are ubiquitously active are used for gene expression in human cells. However, viral promoters are often inactivated in eukaryotic cells due to hypermethylation [38,39]. For long-term expression of transgenes in human cells, eukaryotic or eukaryotic/viral hybrid promoters are used, which remain active for a long time [40,41]. In addition, cell-type-specific promoters may be used in expression cassettes of DNA vaccines, which can restrict the expression of antigens and genetic adjuvants to APC cells such as dendritic cells (DC), macrophages and B cells. The restriction of gene expression to APCs prevents tolerance induced by regulatory cells such as myeloid-derived suppressor cells (MDSC) and Tumor-associated macrophages (TAM). The promoter of the gene encoding for Fascin-1 is a DC-specific promoter which is highly expressed in activated DC cells as well as in neuronal cells in humans and mice. Immunization of mice with DNA vaccines containing the Fascin-1 promoter activated Th1-biased immune responses and cytotoxic T cells (CTL). However, transcriptional targeting of DC with the fascin-1 promoter also eliminates antigen expression in B cells, which may impair the induction of humoral immune responses [27,42–49]. Alternatively, APCs can be targeted *ex vivo*, in which the cells are isolated from the body, transfected with DNA vaccine *in vitro*, and then injected back into the body [50,51].

## Production of DNA vaccines

Different DNA constructs, including circular DNA constructs such as plasmid DNA [52] and minicircle DNA [53,54], or linear, covalently-closed minimalistic expression constructs such as MIDGE [55,56] and Doggybone™ [57–59], are produced by different methods.

**Figure 1.**
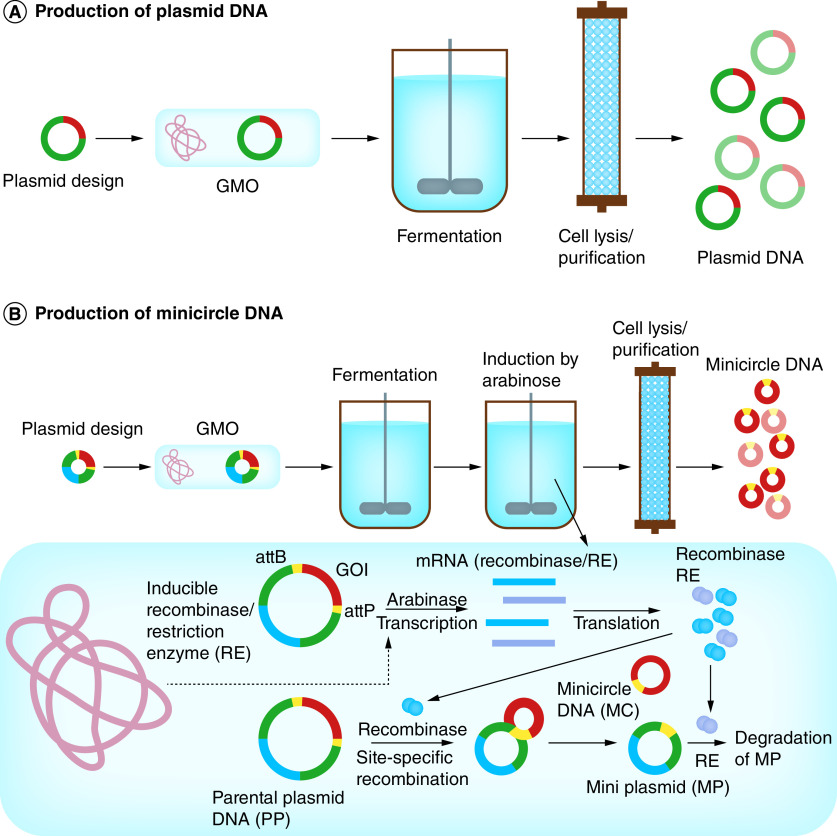
Production of circular DNA constructs. **(A)** Production of plasmid DNA. The designed plasmid containing the GOI is transformed to the bacterial host to generate a GMO. After fermentation, bacterial cells are harvested and lysed through chemical, physical or mechanical methods. After the removal of solids from the lysate (clarification), several steps of purification such as chromatographic purification are used for the purification of plasmid DNA. **(B)** Production of minicircle DNA. After the growth of the genetically modified bacteria containing the parental plasmid, the expression of recombinase and the restriction enzyme is induced by arabinose from the plasmid or bacterial genome. The recombinase initiates the site-specific recombination between its recognition sequences (attB and attP), originating the minicircle DNA and a MP consisting of the bacterial backbone. Then, MP is degraded specifically by the RE. The minicircle DNA is extracted and purified after cell harvest, cell lysis, clarification and several steps of purification. GOI: Gene of interest; GMO: Genetically modified organism; MP: Mini plasmid; RE: Recombinase.

Plasmid DNA is produced through genetically modified bacteria, usually *Escherichia coli* (Figure 1 A). The good manufacturing process (GMP)-production of plasmid DNA at preclinical and clinical scale requires careful development of optimal and economical commercial processes [60]. Bacterial cells are grown under fermentation conditions usually in defined or minimal cell culture media consisting of chemically defined substances such as glucose or glycerol as carbon sources, salts, vitamins, etc. After fermentation, bacterial cells are harvested by centrifugation or microfiltration. Then chemical, physical or mechanical methods are employed for cell lysis. The lysate generated by cell lysis contains cell debris, plasmid DNA and soluble impurities. Clarification techniques such as cross-flow microfiltration are used to remove solids from the lysate. Then, several steps of purification such as contaminant precipitation, plasmid precipitation, chromatographic purification (anion exchange chromatography, followed by hydrophobic interaction chromatography and sometimes by size exclusion chromatography) are employed for the removal of contaminants (e.g., host proteins, endotoxins, RNA, genomic DNA and linear and open-circular forms of plasmid DNA). The purified plasmid DNA is formulated with excipients and adjuvants and filtered through sterilizing filters such as polyethersulfone (PES) and polyvinylidene difluoride (PVDF) membranes [52].

Minicircle DNA is produced by inducing the intramolecular recombination of a parental plasmid in *E. coli*. For example, the expression of a recombinase such as ϕC31 integrase and a restriction enzyme (RE) such as I-SceI are induced by an arabinose-inducible gene expression system. The recombinase mediates the site-specific recombination between its recognition sequences (e.g., attB and attP), originating two different circular DNA molecules (i) the minicircle DNA containing the eukaryotic expression cassette and (ii) a mini plasmid (MP) consisting of the bacterial backbone. MP can be degraded specifically by the induced RE. The minicircle DNA can then be extracted, purified and formulated similar to the methods used for DNA plasmids (Figure 1 B) [53,54].

**Figure 2.**
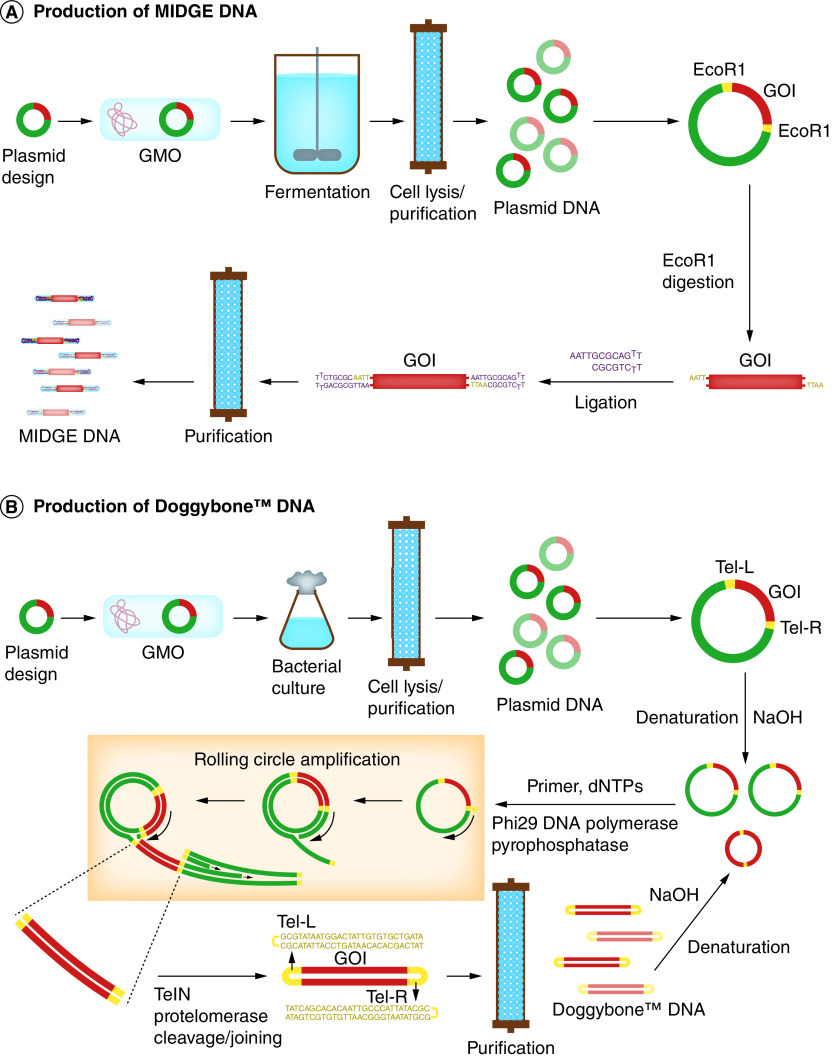
Production of linear, covalently-closed minimalistic expression DNA constructs. **(A)** Production of MIDGE DNA. The purified parental plasmid containing the GOI flanking with a restriction site (e.g. EcoRI) is digested by the RE. The resulting fragments are ligated to the hairpin oligodeoxynucleotides to generate MIDGE DNA molecules. Then, unligated fragments including plasmid backbones are digested by T7 DNA polymerase and the MIDGE DNA is purified. **(B)** Production of Doggybone™ DNA. The purified parental plasmid containing the GOI flanking with telomeric ends (Tel-L and Tel-R) is denatured by NaOH and used as a template DNA in a RCA reaction using Phi29 DNA polymerase. The resulting DNA concatemers are cleaved and joined by an enzymatic reaction using TelN protelomerase to generate Doggybone DNA. Doggybone™ DNA molecules are then purified using chromatographic purification. The purified Doggybone DNA may be used as a template DNA for the RCA reaction. GMO: Genetically modified organism; GOI: Gene of interest; MIDGE: Minimalistic, immunologically defined gene expression; RCA: Rolling circle amplification.

MIDGE vectors are produced by digestion of DNA plasmid using an RE such as EcoRI, and subsequent ligation of the resulting fragments to hairpin oligodeoxynucleotides to generate a covalently closed dumbbell-shaped DNA molecule (Figure 2 A). Unligated fragments including plasmid backbones are digested by T7 DNA polymerase. MIDGE vectors can be further purified by chromatographic purification (e.g., anionic-exchange chromatography) and formulated similar to DNA plasmids [55,56].

Doggybone™ DNA is produced through an enzymatic process (Figure 2B). The DNA plasmid containing the eukaryotic expression cassette flanking with telomeric ends (Tel-L and Tel-R) is denatured by NaOH and used as a template DNA in a rolling circle amplification (RCA) process. The resulting double-stranded DNA concatemers are cleaved and joined by TelN protelomerase to generate the linear, covalently closed, double-stranded molecules. Doggybone™ DNA molecules are purified using chromatographic purification and formulated similar to DNA plasmids [57–59].

## Adjuvants

Vaccine adjuvants may be used for improving the immunogenicity of DNA vaccines by stimulating innate immune responses. Plasmids are usually produced in bacterial hosts and thus have unmethylated CpG motifs, which may have an intrinsic adjuvant effect by stimulating innate immune responses through TLR9 [61]. However, different types of adjuvants have been used with DNA vaccines in exploratory and preclinical studies including classical adjuvants liposomal and nanoparticle adjuvants, and molecular adjuvants [62,63].

When using classical adjuvants such as aluminum salts (alum) in vaccination, in many cases, antigen and adjuvant are mixed before administration, allowing the physical interaction of adjuvant and antigen. This may lead to slower antigen release and longer interaction with immune cells. While several classical adjuvants have been used with DNA vaccines in animal models, no benefits have been found in large animals such as nonhuman primates in comparative studies [61]. The positive effect of such adjuvants in small animals (e.g., mice) has been attributed to the boosting of immune responses elicited by the expressed antigen rather than to the physical interaction of adjuvants and DNA vaccines [61]. In humans, aluminum phosphate adjuvant did not show any significant effect on the immunogenicity of a DNA vaccine for human immunodeficiency virus (HIV) [64].

Different types of adjuvants have been used with DNA vaccines for veterinary applications [65]. Some of these adjuvants have already been licensed for veterinary use. West Nile-Innovator^®^ DNA is a WNV DNA vaccine approved in 2005 for veterinary use in horses, containing a plasmid DNA encoding antigenic proteins of WNV and a lipid-based adjuvant MetaStim™ [66]. West Nile-Innovator DNA vaccine was removed from the market in 2010 [66]. AgriLabs ExactVac is the first commercial DNA vaccine against the H5N1 influenza virus in chickens. This vaccine has been formulated with a lipid/polymer matrix-based adjuvant named ENABL^®^. It is believed that ENABL enables efficient dispersion of the vaccine micro-particles and more efficient delivery of the vaccine to target cells [67]. However, it has not been reported whether MetaStim and ENABL adjuvants improved the immunogenicity of these DNA vaccines.

Nanoparticles such as liposomes, PLGA and exosomes can boost immune responses even in the empty form. Therefore, nanoparticles may be used as adjuvants in vaccine formulations [68]. Nanoparticles can protect DNA from degradation and therefore can enhance the immune response compared with naked DNA vaccines [69]. Different types of nanoparticles such as cationic liposomes [70], magnesium phosphate nanoparticles [71] and calcium phosphate nanoparticles [72] have been used as adjuvants in DNA vaccine formulations to improve the immunogenicity of DNA vaccines in animal models.

Molecular adjuvants comprise signaling molecules such as toll-like receptor (TLR) agonists, chemokines, cytokines, immune costimulatory molecules and inhibitors of immune-suppressive signaling pathways. The encoding genes for molecular adjuvants could be incorporated into the sequence of DNA vaccines with a eukaryotic expression cassette [62,63].

In recent years, some molecular adjuvants formulated with DNA vaccines have undergone clinical trials in humans, either as immunostimulatory sequences fused to the sequence of the target antigen (e.g. human chemokine CCL20 (MIP3α) and potato virus X coat protein (PVXCP)) [73] or encoded by separate plasmids (e.g. DNA plasmids encoding human cytokines such as IL-2, IL-12, IL-15, IFN-lambda 3 and granulocyte-macrophage colony-stimulating factor (GM-CSF)) [74–77].

## Delivery & administration routes of DNA vaccines

The route of DNA vaccine administration may influence its immunogenicity. Various administration routes such as intramuscular (IM), intradermal (ID), subcutaneous (SC), intravenous (IV), intranodal and intranasal routes have been used to elicit a desired immune response after DNA vaccination. In the first study of DNA vaccines, the vaccine was administrated to mice via the IM route [4]. However, subsequent studies showed that the ID route may increase the expression and immunogenicity of DNA vaccines in mice compared with the IM route [78–80]. Immunization of the skin with DNA vaccines provides DNA for antigen expression in several types of cells including Langerhans cells, dendritic cells and keratinocytes, which are located in the epidermis and the dermis layers, the two main areas of the skin. After maturation, dermal dendritic cells and Langerhans cells can migrate to local lymph nodes and present antigens to T cells, thus starting a variety of immune responses [78]. In addition, SC injection of DNA under high pressure has been reported to evoke greater immune responses in mice than by the IM route [81,82]. Mucosal immunization with DNA vaccines through oral or nasal delivery is another route of delivery for DNA vaccines that generates mucosal as well as systemic immune responses [83,84].

**Figure 3.**
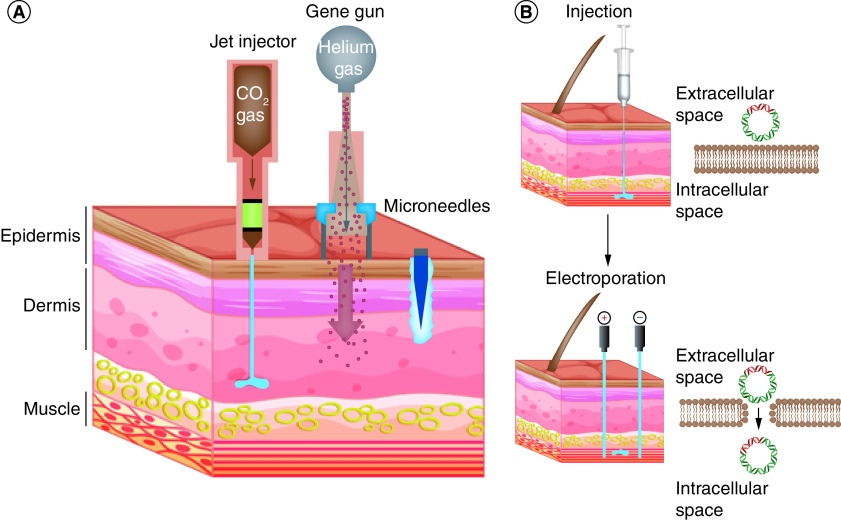
Overview of physical DNA vaccine delivery technologies. **(A)** Delivery of DNA vaccines into skin compartments using gene gun, jet injector and microneedles. These physical methods allow for delivery of DNA vaccines into the epidermis, dermis and subcutaneous compartments by providing enhanced efficacy and great safety than conventional needle methods. **(B)** The use of *in vivo* electroporation enhances the cellular uptake of injected DNA across the cell membrane.

In addition to the administration route, there are multiple physical and chemical methods to increase the efficiency of DNA delivery into cells both *in vitro* and *in vivo* and increase its immunogenicity when used as a vaccine [80]. In jet-injector (biojector)-based delivery, usually compressed CO2 gas is used to create a high-pressured stream of medications such as DNA vaccine that can penetrate the skin and elicit higher cellular immunity and antibody responses in humans compared with the conventional syringe and needle vaccine delivery (Figure 3A) [85]. In gene gun delivery, a biolistic system is used that can push DNA-coated microparticles (e.g., DNA-coated gold particles) directly into the skin (Figure 3A). One of the advantages of this method is that lower amounts of DNA are needed to elicit an immune response compared with conventional injection [86]. In the microneedle array-based delivery, over 1000 microneedles (usually 100–1000 μm in length) are used to inject medications such as DNA vaccine with a direct and controlled route to the underlying viable skin layers (Figure 3A) [87,88]. Electroporation is another method for DNA delivery that uses electrical pulses to create transient pores in the cell membrane, thereby increasing DNA uptake (Figure 3B) [89,90]. In humans, *in vivo* electroporation of DNA for IM delivery elicited a greater magnitude of HIV-specific cellular immunity compared with the traditional syringe and needle IM delivery [91]. The growing number of clinical trials in humans and the corresponding results showed the strong potential of electroporation for DNA vaccination, which combines both efficacy and safety [92]. In addition to these physical methods, liposomes, virosomes, and other synthetic and natural microparticles and nanoparticles such as Fe_3_O_4_, polyethyleneimine (PEI), polyamidoamine (PAMAM) and poly(propyl ether imine) (PETIM) dendrimers, chitosan, alginate, dextran, chondroitin sulfate, hyaluronic acid, pullulan, Gelatin, albumin, listeriolysin O (LLO), protamine, epsilon poly-L-lysine, pectin, zein, polyspermine, polyarginine, polydopamine (PDA), polyglutamate (PGA), poly-lactic acid (PLA), poly(lactic-glycolic acid) (PLGA) agarose hydrogel, spermine dextran, cell penetrating peptides (CPPs), poly(beta-amino esters) (PBAE), acrylamide microspheres and protein-based nanoparticles may be employed for DNA vaccine delivery. Such micro- and nanoparticles protect DNA from degradation by nucleases in the body and also increase the cellular uptake of DNA vaccines through endocytosis, thereby improving the immunogenicity of DNA vaccines [80,93–96].

**Figure 4.**
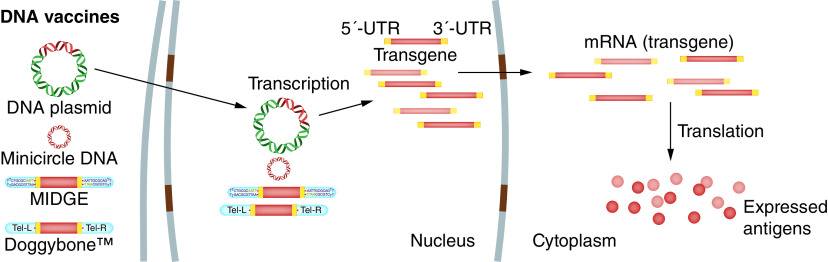
Antigen expression by DNA vaccines. The DNA construct encoding the transgene (vaccine antigen) translocates to the nucleus, where the transcription to mRNA takes place. Produced mRNAs are then delivered to the cytoplasm to ensure efficient translation of the vaccine antigen. MIDGE: Minimalistic, immunologically defined gene expression.

**Figure 5.**
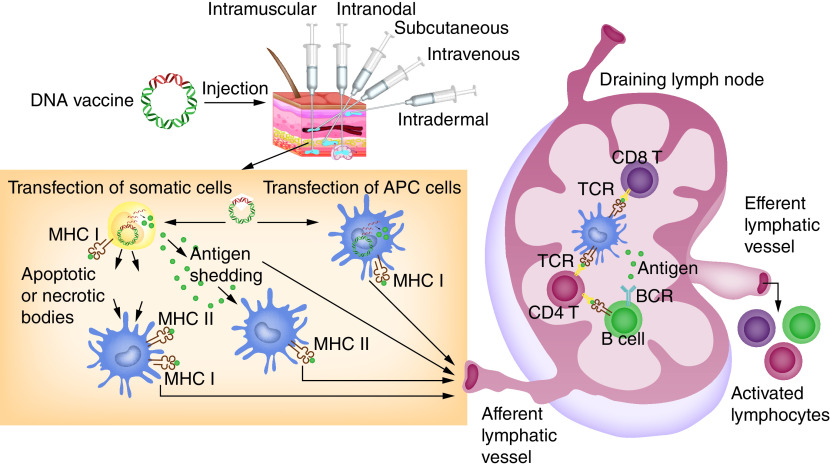
Induction of adaptive immune responses by DNA vaccines. DNA vaccines are administered by multiple routes. The injected DNA enters the somatic cells and/or APCs to produce target antigens, which is the subject of immune surveillance by both MHC class I and class II molecules of APCs. Following the presentation of antigens, activated APCs travel to the draining lymph nodes to present antigenic peptides to CD4 and CD8 T cells. This interaction allows activation and expansion of T cells, as well as antibody-producing B cells. Both humoral and cellular immune responses are induced to trigger a robust response against the target antigens. APCs: Antigen-presenting cells; BCR: B-cell receptor; MHC: Major histocompatibility complex; TCR: T-cell receptor.

After the DNA vaccine is administrated to the inoculation site using one of several delivery methods, the plasmid translocates to the nucleus of transfected APCs or somatic cells and begins transcription, followed by protein production in cytoplasm and the formation of foreign antigens (Figure 4). After being expressed in APCs and somatic cells, the antigens are taken up by APCs and processed to small peptides which can be displayed on MHC I or II molecules that activate CD8 cytotoxic T lymphocytes (CTLs) and CD4^+^ T helper (T_h_) cells, respectively. In addition, B lymphocytes capture antigens released from transfected somatic cells such as myocytes and keratinocytes, which activate humoral immunity (Figure 5) [97,98].

## DNA vaccines for COVID-19

The preclinical studies and clinical trials of DNA vaccines for infectious diseases such as influenza virus, HIV, cytomegalovirus (CMV), human hepatitis virus C (HCV), Venezuelan equine encephalitis virus (VEEV), Zika virus (ZIKV), Ebola virus (EBOV) and Middle East respiratory syndrome coronavirus (MERS-CoV) as well as for immunotherapy of viral diseases and cancer have been previously reviewed [3,98]. In this section, we focus on preclinical and clinical trials of DNA vaccines for COVID-19 disease caused by SARS-CoV-2.

The global vaccine research and development (R&D) for the pandemic COVID-19 is unprecedented in scale and speed. Due to the need for speed, vaccine designers and developers are making a fundamental change in the vaccine production process, which previously took more than 10 years, even compared with the 5-year acceleration time scale for the production of the first Ebola vaccine. This accelerated process requires a new paradigm of vaccine development involving parallel development phases, new regulatory processes and manufacturing capacity scaling [99]. Although DNA vaccines have a new platform technology that was not previously available on the market for human use, they are very promising in the SARS-CoV-2 vaccine race [100,101]. DNA vaccines can be designed and produced within days after obtaining the genome sequence of the pathogen or nucleotide sequence of cancer antigens. Plasmid DNA manufacturing processes allow for scalable manufacture of DNA vaccines, making them ideal for rapid control of newly emerging pathogens, which circumvent the problems of conventional vaccines produced in eggs or eukaryotic cell culture bioreactors [102,103]. However, the novel vaccine technology platforms such as DNA vaccines for a new virus target, and novel development paradigms may increase the risks associated with injecting an approved vaccine, requiring careful evaluation of safety and effectiveness at each step of vaccine development. Scientists have developed specific animal models such as human ACE2 (hACE2) expressing transgenic mice, hamsters and non-human primates to evaluate the protective efficacy of anti-COVID-19 vaccines in preclinical studies [99]. Another suitable animal model involves the intranasal delivery of a viral vector encoding hACE2 to wild-type animals prior to viral challenge [104]. Several preclinical studies and clinical trials have demonstrated the immunogenicity of DNA vaccines against SARS-CoV-2, which are summarized in Tables 1 and 2, respectively. Preclinical experiments demonstrated that DNA vaccines can elicit both humoral and cellular immune responses in animal models (Table 1).

**Table 1.** Overview of published preclinical *in vivo* studies of DNA vaccines against SARS-CoV-2.

Category	Animal model	Delivery route	Dose	Antigen	Immune responses	Ref.
Plasmid DNA	BALB/c mice, C57BL/6 mice, and guinea pigs	IM + EP for mice, ID + EP for guinea pigs	Twice (2.5, 10 or 25 μg) for mice, single (100 μg) for guinea pigs	S protein	H, C, nAb	[103]
Plasmid DNA	Rhesus macaques	IM	Twice (5 mg)	Different forms of the S protein: full length, deletion of the cytoplasmic tail, the soluble ectodomain, S1 domain with a foldon trimerization tag, RBD with a foldon trimerization tag, prefusion-stabilized soluble ectodomain with two proline mutations, deletion of the furin cleavage site and a foldon trimerization tag	H, C, nAb	[105]
Plasmid DNA	BALB/cJ mice	EP	Twice (60 μg)	Engineered RBD, with four novel glycosylation sites, fused to multimerization platforms	nAb	[106**]**
Plasmid DNA	Syrian hamsters	IM + jet injection	Single (0.2 mg)	S protein	nAb	[107**]**
Plasmid DNA	C57BL/6 and BALB/c mice	(IM or ID) + EP	Twice (days 1 and 14 or 21)	Prefusion-stabilized S protein/alone or combined with plasmid IL-12	nAb	[108**]**
Plasmid DNA	Rhesus macaques	Needle Free Injection System (NFIS)/Syringe-needle injection (ID)	Thrice (days 1, 28 and 56)	S protein	nAb	[109**]**
Plasmid DNA	C57BL/6 mice	IM	Thrice (weeks 0, 2 and 4)	RBD fused to the amino-terminal region of hepatitis B virus preS1 with a W4P mutation	H, C, nAb	[110**]**
Plasmid DNA	ICR mice	IM + EP	Thrice (weeks 0, 2 and 4)	S protein or S1 subunit or S2 subunit	H, C, nAb	[111**]**
Plasmid DNA+ recombinant protein	Rhesus macaques	IM	Thrice (weeks 0, 2 and 8)	S protein (Plasmid DNA) + S1 subunit (Recombinant protein)	nAb	[112**]**

C: Cellular immune response; EP: Electroporation; H: Humoral immune response; ID: Intradermal; IM: Intramuscular; nAb: Neutralizing antibody; RBD: Eeceptor-binding domain; S: Spike protein.

**Table 2.** Overview of clinical trials of DNA vaccines against COVID-19 based on WHO report: draft landscape and tracker of COVID-19 candidate vaccines – 4 May 2021 (https://www.who.int/publications/m/item/draft-landscape-of-covid-19-**candidate-vaccines**).

Vaccine type (vaccine name)/description	Developer	Participants (n)	Dose/route	Clinical stage, outcome	Trial registration no.	Ref.
Plasmid DNA (INO-4800)/a pGX9501 plasmid that encodes for the full length of the S protein	Inovio Pharmaceuticals/ International Vaccine Institute	40 healthy adults (18–50 years)	2× (1 or 2 mg) on day 0 and day 28/ID injection followed by EP using the CELLECTRA^®^ 2000 device	I – (INO-4800 was safe and immunogenic in all of the vaccinated subjects. The vaccine elicited either or both humoral or cellular immune responses)	NCT04336410	[16,19]
Plasmid DNA (INO-4800)	Inovio Pharmaceuticals/ International Vaccine Institute	160 healthy adults (19 years and older)	2× (1 or 2 mg)/ID injection followed by EP using the CELLECTRA^®^ 2000 device	I/II	NCT04447781	
Plasmid DNA (INO-4800)	Inovio Pharmaceuticals/ International Vaccine Institute	640 healthy adult (18–60 years) and elderly (60–85 years) volunteers	2× (ID + EP)	II	ChiCTR2000040146	
Plasmid DNA (INO-4800)	Inovio Pharmaceuticals/ International Vaccine Institute	6578 healthy adults (18 years and older)	1× or 2× (1 mg) on day 0 and day 28/ID injection followed by EP using the CELLECTRA^®^ 2000 device	II/III	NCT04642638	[18]
Plasmid DNA (AG0301-COVID19)/a Plasmid DNA + adjuvant	AnGes + Takara Bio + Osaka University	30 healthy adults (20 years and older)	2× (1 or 2 mg)/IM	I/II	NCT04463472	
Plasmid DNA (AG0301-COVID19)	AnGes + Takara Bio + Osaka University	500 healthy adults (18 years and older)	2× 2 mg/IM	II/III	NCT04655625	
Plasmid DNA (AG0302-COVID19)/a Plasmid DNA + adjuvant	AnGes + Takara Bio + Osaka University	30 healthy adults (20 years and older)	2× or 3× 2 mg/IM	I/II	NCT04527081	
Plasmid DNA (nCov Vaccine/ZyCOV-D)	Zydus Cadila	1048 (18–55 years of age in Phase I, ≥12 years of age in Phase II)	3× (1 or 2 mg)/ID injection by needle or PharmaJet^®^	I/II	CTRI/2020/07/026352	
Plasmid DNA (nCov Vaccine/ZyCOV-D)	Zydus Cadila	150 healthy subjects (18–60 years)	2× (3 mg)/ID injection by PharmaJet^®^	I/II	CTRI/2021/03/032051	
Plasmid DNA (Covigenix VAX-001)/DNA vaccines + proteo-lipid vehicle formulation	Entos Pharmaceuticals Inc.	72 healthy adults (18–84 years)	2× IM on day 0 and day 14	I	NCT04591184	
Plasmid DNA (CORVax 12)/encoding SARS-CoV-2 S protein with or without the combination of IL-12p70 plasmid	Providence Health & Services	36 healthy adults (18 years and older)	2× ID followed by EP on day 0 and day 28	I	NCT04627675	
Plasmid DNA (GLS-5310)	GeneOne Life Science, Inc.	345 healthy adults (19–65 years)	2× (0.6 or 1.2 mg)/ID on day 0 + 56 or day 0 + 84	I/II	NCT04673149	
DNA (GX-19)/DNA vaccine encoding SARS-CoV-2 S protein	Genexine Consortium	210 healthy adults (18–50 years)	2× IM injection via EP or PharmaJet^®^	I/II	NCT04445389	[17]
DNA (GX-19N)/DNA vaccine encoding SARS-CoV-2 S protein antigen including the Nucleocapsid protein (NP) antigen	Genexine Consortium	170 healthy adults (18–55 years)	2× IM injection via EP	I/II	NCT04715997	[17]
DNA (COVIGEN)	University of Sydney, Bionet Co., Ltd Technovalia	150 healthy adults (18–75 years)	2× (IM or ID)	I	NCT04742842	
Plasmid DNA (COVID-eVax)/DNA vaccine encoding SARS-CoV-2 S protein	Takis + Rottapharm Biotech	160 healthy adults (18–65 years)	2× (0.5, 1 or 2 mg) IM injection via EP	I/II	NCT04788459	

EP: Electroporation; ID: Intradermal; IM: Intramuscular; RBD: Receptor-binding domain; S: Spike.

The spike (S) protein of SARS-CoV-2 is the main antigen used in preclinical and clinical trials of DNA vaccines for COVID-19 (Tables 1 and 2). S protein has two subunits, S1 and S2. S1 subunit has a receptor-binding domain (RBD) that binds to the human ACE2 receptor and attaches virus particles to the host cell membrane, initiating the infection process [113]. The S2 subunit contains a fusion peptide that helps the fusion of viral and host cell membranes during the process of virus entry into the host cell [113]. Different forms of the S protein, including the full-length S protein (either wild-type or a prefusion-stabilized version), RBD, S1 subunit, and S2 subunit are currently used in preclinical and clinical trials of DNA vaccines for COVID-19 (Tables 1 and 2). Nucleocapsid (N) protein is another antigen used in a SARS-CoV-2 candidate DNA vaccine named GX-19N (ClinicalTrials.gov number, NCT04715997) along with the S protein (Table 2). N protein protects the viral genome and is also involved in the release of viral particles from infected cells [113].

The IM and ID routes of administration are the main routes of DNA delivery in both preclinical and clinical trials of DNA vaccines for COVID-19 (Tables 1 and 2). In addition, EP and jet-injection are the main physical methods to improve the efficiency of DNA vaccine delivery in both preclinical and clinical trials of COVID-19 (Tables 1 and 2).

There are currently 11 candidate DNA vaccines in clinical trials for COVID-19 (Table 2). INO-4800 is a pGX9501 plasmid that encodes the full length of the SARS-CoV-2 S protein. In a Phase I clinical trial (ClinicalTrials.gov number, NCT04336410), INO-4800 was injected into 40 healthy adults aged between 18 and 50 years in two doses (1 or 2 mg of plasmid DNA at day 0 and day 28) by ID injection followed by EP using the CELLECTRA^®^ 2000 device [16]. INO-4800 elicited adequate humoral responses against SARS-CoV-2. The vaccine induced binding or neutralizing antibodies in 95% (18/19) of the participants in both dose groups. Neutralizing antibodies were seen in 78% (14/18) and 84% (16/19) of the participants in the 1- and 2-mg dose groups, respectively. The corresponding geometric mean titers (GMTs) were 102.3 (95% CI: [37.4, 280.3]) for the 1-mg dose group and 63.5(95% CI: [39.6, 101.8]) for the 2-mg dose group based on the plaque-reduction neutralization testing (PRNT) assay with live SARS-CoV-2, at day 42 [16]. The range of GMTs overlaps with the PRNT IC_50_ titers reported from convalescent patients and meets the US FDA recommended (160) and minimal (80) GMT for convalescent plasma use [114]. In addition, good CD4 and CD8 T-cell responses were observed in the trial, especially in the 2 mg-dose group. T-cell responses were activated in 74 and 100% of the 1- and 2-mg dose groups, respectively. However, cellular immune responses were higher in the 2-mg dose group than in the convalescent samples. According to the report, INO-4800 was safe and immunogenic in all of the vaccinated individuals [16]. The antibody response persisted 6 months following the second dose of INO-4800 vaccine, and a booster dose 6–10.5 months following the second dose significantly increased immune responses [19]. In a Phase II clinical trial (ClinicalTrials.gov number, NCT04642638), INO-4800 was evaluated in 401 participants in two doses (1 or 2 mg of plasmid DNA at day 0 and day 28) by ID injection followed by EP using the CELLECTRA^®^ 2000 device. At day 42, the GMT (SD of log10) of neutralizing antibody in the 1- and 2-mg dose groups were 93.6 (0.47) and 150.6 (0.46), respectively. The baseline GMTs for the 1- and 2-mg dose groups were 32.2 (0.38) and 35.8 (0.45), respectively. Based on this clinical trial, humoral and cellular immune responses were higher in the 2-mg dose group compared with the 1-mg dose group and thus INO-4800 2-mg dose was selected for advancement into a Phase III efficacy evaluation [18].

Based on a pseudovirus neutralization assay using sera collected from INO-4800 vaccinated individuals two weeks after administration of a third dose (0.5 mg, 1 mg, or 2 mg; NCT04336410), there was a 2.1- and 6.9-fold reduction of neutralizing activity against SARS-CoV-2 variants B.1.1.7 (first reported in the UK) and B.1.351 (first reported in South Africa), respectively, while there was no difference between P.1 (first reported in Brazil) variant and wild-type (WT). Surprisingly, despite recent studies indicating a reduction in neutralizing activity against SARS-CoV-2 variant P.1, INO-4800 vaccine generated neutralizing antibodies at levels comparable to the WT. INO-4800 cellular immune response was similar against these variants and WT [115].

GX-19 is a DNA vaccine that contains a plasmid DNA encoding SARS-CoV-2 S protein, and GX-19N contains a plasmid DNA encoding SARS-CoV-2 RBD and N protein as well as a plasmid DNA encoding SARS-CoV-2 S protein. In two Phase I trials of GX-19 and GX-19N (NCT04445389 and NCT04715997), GX-19 and GX-19N vaccines were evaluated in 40 and 21 participants, respectively [17]. Two doses (1.5 mg or 3 mg of plasmid DNA for GX-19 and 3 mg for GX-19N at day 0 and day 28) were injected by the IM route followed by EP. GX-19 and GX-19N showed low GMTs of neutralizing antibodies. In GX-19N group, neutralizing antibodies significantly increased after vaccination, but the GMT of neutralizing antibodies on day 57 (37.26) was lower than those from human convalescent serum. However, GX-19 and GX-19N showed significantly enhanced T-cell responses. S-specific T-cell responses were seen in 50% (10/20) of the participants in both dose groups in the GX-19 trial. GX-19N vaccine induced stronger T cell immune responses than GX-19 vaccine and exhibited S- and N-specific T-cell responses. T-cell responses were seen in 75% (15/20) of the participants in the GX-19N trial [17].

nCov Vaccine (ZyCoV-D) is a DNA vaccine that contains a plasmid DNA encoding SARS-CoV-2 S protein. In a Phase I clinical trial, the safety and immunogenicity of ZyCoV-D was evaluated in 126 participants. Three doses of ZyCoV-D (1 mg or 2 mg) were administrated by the ID route via NFIS device 28 days apart. ZyCoV-D was found to be safe and immunogenic in the Phase I trial. However, the GMTs of neutralizing antibodies were low (<40). Based on the ELISPOT assay, ZyCoV-D vaccine induced cellular immune responses when administered ID via NFIS at 2-mg dose. However, there were no significant changes in IFN-γ, IL-2, IL-4, IL-6, IL-10, TNF-alpha and Th-17A cytokines levels compared with baseline [15]. Based on the unpublished results of a Phase III clinical trial, ZyCoV-D has been found to be 67% protective against symptomatic COVID-19 [116]. India's drug regulator has approved ZyCoV-D vaccine for emergency use against COVID-19 [116]. ZyCoV-D is the world's first approved DNA vaccine to be administered in humans [117].

## Advantages & obstacles of DNA vaccines

DNA vaccines hold several advantages that can make them a suitable vaccine against newly emerging pathogens such as SARS-CoV-2 virus: i) The manufacturing of DNA vaccines is inexpensive, rapid and scalable. A bacterial culture fermenter is used to provide large-scale plasmid DNA. Initial plasmid construction, cell bank preparation, bacterial fermentation and plasmid purification are completed within 2–4 weeks [60]; ii) DNA vaccines have fast and flexible R&D. They can be rapidly designed and produced for preclinical studies and clinical trials; iii) DNA vaccines express viral and cancer antigens matching better with their native form. DNA vaccines deliver genetic material into a host cell and use the host cell translational machinery to express protein antigens in their native folding and glycosylation pattern, without the problems commonly associated with protein expression and purification issues such as protein solubility in the production of recombinant proteins [100]; IV) DNA vaccines generate foreign intracellular antigens which are presented in the context of MHC class I as well as class II molecules of APCs, eliciting both cellular and humoral immune responses; V) DNA vaccines are relatively stable at ambient temperatures; VI) DNA vaccines can simply be equipped with an additional expression cassette encoding a molecular adjuvant to elicit stronger immune responses; VII) unlike viral vector vaccines, pre-existing immunity to the vaccine backbone is not a problematic factor for DNA vaccines due to the lack of a host immune response to plasmid DNA [118].

However, in the application of DNA vaccines, some obstacles may need to be overcome: i) Once administered, naked DNA vaccines are rapidly degraded by nucleases in the mucosa, skin, and plasma, and only small amounts of injected DNA are taken up by cells, resulting in low efficiencies; ii) different biological barriers including cell membrane, endosomes and nucleus membrane are barriers for the DNA vaccines reaching their target site; iii) after reaching the cell nucleus, low antigen expression is another barrier that may lead to low immunogenicity of DNA vaccines; IV) DNA vaccines may continuously express the target antigen, which may lead to potential tolerance to the pathogen [119].

Improving delivery and antigen expression may improve the performance of DNA vaccines [120]. Delivery systems can increase the delivery of DNA vaccines to reach their target (cell nucleus) and/or protect them from degradation by extracellular nucleases. In the development of DNA vaccines for COVID-19, physical methods (e.g., electroporation and jet-injection) are the most widely used delivery systems for delivering DNA vaccines to the required intracellular location (Table 2). DNA vaccines are stable and therefore usually administered in naked form. But milligram amounts of a DNA vaccine are required to be injected into a human to elicit potent immune responses. This is a barrier to success in industrial production of DNA vaccines as that amount of DNA is expensive to produce. Physical methods such as electroporation, jet-injection and gene gun can reduce the amount of DNA needed for immunization and increase delivery efficiency. But specialized devices are needed for *in vivo* electroporation, jet-injection and gene gun-mediated delivery of DNA vaccines in research and in the hospital setting. Several types of natural and synthetic nanoparticles may be used for DNA vaccine delivery and protection from degradation. Although DNA plasmids can be integrated into the genome of host cells, the level of integration is relatively low [121]. Six weeks after intramuscular injection of three different DNA plasmids in mice, the level of free plasmids in the treated muscle ranged from 1000 to 4000 copies/μg of host DNA (1 μg of DNA represented ∼150,000 diploid cells). After six months, the free plasmids were still stable in muscle, in the range of 200–800 copies/μg of DNA, and no integration of the DNA plasmid to genomic DNA was observed [122]. Therefore, it is believed that the risk of insertional mutations due to the integration of DNA plasmids following injection is negligible and integration is usually done at rates that are less than the frequency of the spontaneous mutations [97]. In contrary, there are some reports of leukemogenesis driven by viral vector insertional mutagenesis in severe combined immunodeficiency (SCID) gene therapy clinical trials in humans [123,124]. In addition, linearized plasmids have an increased probability of chromosomal integration [125]. Therefore, there is a potential risk of insertional mutagenesis following mechanical shearing of plasmid DNA. The FDA designed guidelines for DNA vaccines to ensure that the frequency of plasmid integration would be lower than the spontaneous mutation rate [126]. Based on these guidelines, we can monitor the biodistribution of plasmids in tissues of vaccinated animals by sensitive q-PCR assays. The integration of plasmids can be assessed by separating high molecular weight genomic DNA from smaller free plasmids. The q-PCR and/or repeat-anchored integration capture (RAIC)-PCR techniques are then used to detect and quantify the integration of plasmid in the genomic DNA. Based on studies on multiple different plasmids, and of the same plasmid with various DNA inserts, FDA proposed that DNA vaccines prepared using a plasmid DNA previously documented to have an acceptable DNA biodistribution/integration profile could waive biodistribution/persistence studies. Integration studies are required for novel plasmids and novel methods of formulation and delivery for plasmids that persist at amounts of higher than 10,000 copies per microgram of host DNA [126]. Recently, the FDA paused the planned Phase II/III trial of the vaccine candidate INO-4800 due to questions about the design and use of the INOVIO delivery machine ‘CELLECTRA^®^ 2000’, which is used to deliver the vaccine directly into the skin [127]. Less than two months later, the FDA allowed INOVIO to move forward with the Phase 2/3 trial (clinical trial identifier: NCT04642638) [127]. Given that free plasmids remain stable in the host cell for months, the question of whether prolonged expression of antigen by DNA vaccines can lead to tolerance requires further investigation.

## Conclusion

Over the past few years, many advances have been made in the field of DNA vaccines. The advancements in DNA construction, delivery and administration routes and use of molecular adjuvants have enhanced the immunogenicity of DNA vaccines. It is promising that DNA immunization will revolutionize the vaccine field. The lower cost of manufacturing and storage of DNA vaccines makes them an ideal candidate vaccine for global vaccination, even in low-income countries. This new vaccine platform is now very promising in clinical trials for COVID-19, and scientists are trying to get the first DNA vaccine license for humans.

## Future perspective

In the future, we could devise new platforms of DNA vaccines such as minicircle DNA, minimalistic, immunologically defined gene expression, and Doggybone™ for DNA vaccination. The use of these new platforms may lead to more transgene expression *in vivo*. Successful DNA delivery and the use of adjuvants remain key challenges in DNA vaccines, especially for large animals and humans, that need to be addressed in the future. We need control groups without receiving adjuvants in clinical trials to accurately assess the efficacy of adjuvants in DNA vaccine formulation. The use of nanoparticles or the design of inexpensive efficient devices for DNA vaccine delivery can facilitate the administration of DNA vaccines in the hospital setting. In addition, we need to examine the impact of different platforms, formulations, storage conditions and DNA delivery methods on the risk of insertional mutations due to DNA integration. The data reviewed here indicate that the DNA platform addresses many goals of a good vaccine platform and will be a new class of future vaccines, especially for emerging pathogens such as SARS-Cov-2.

**Executive summary**

**Design of DNA vaccines**

- DNA vaccines are DNA constructs that contain at least one eukaryotic expression cassette encoding for the antigen of interest.
- Changes in sequence composition and deletion of bacterial backbone sequences in DNA vaccines can increase antigen expression.
- Minicircle DNA, minimalistic, immunologically defined gene expression (MIDGE) and Doggybone™ are DNA constructs composed of the gene expression cassette(s) without the bacterial backbone of plasmids.
- Individual expression cassettes, bi-cistronic or multi-cistronic vectors and T2A peptide sequence can be used to express multiple genes in a single DNA vaccine.
- Nuclear localization signal (NLS) nucleotide sequences, transcription factor-binding proteins, DNA-binding proteins and NLS peptide sequences can be used for enhancing the nuclear localization of DNA vaccines.
- For long-term expression of transgenes in human cells, eukaryotic or eukaryotic/viral hybrid promoters can be used in DNA constructs, which remain active for a long time.
- Cell-type-specific promoters can be used in expression cassettes, which can restrict the expression of antigens to antigen-presenting cells and prevent tolerance induced by regulatory cells.

**Production of DNA vaccines**

- Plasmid DNA is produced through genetically modified bacteria. After fermentation, bacterial cells are harvested and lysed through chemical, physical or mechanical methods. Then, clarification and purification techniques are used for the purification of plasmid DNA.
- Minicircle DNA is produced by inducing the intramolecular recombination of a parental plasmid in bacteria.
- MIDGE vectors are produced by digestion of a plasmid using a recombinase, and subsequent ligation of the resulting fragments to hairpin oligodeoxynucleotides to generate a covalently closed dumbbell-shaped DNA molecule.
- Doggybone™ DNA is produced through RCA process using a DNA plasmid as template. The resulting DNA concatemers are cleaved and joined by an enzymatic process to generate covalently closed dumbbell-shaped DNA molecules.

**Adjuvants**

- Vaccine adjuvants may be used for improving the immunogenicity of DNA vaccines by stimulating innate immune responses.
- Classical adjuvants, nanoparticle adjuvants, and molecular adjuvants have been used with DNA vaccines in exploratory and preclinical studies.
- In humans, aluminum phosphate adjuvant did not show any significant effect on the immunogenicity of a DNA vaccine.
- In recent years, some molecular adjuvants formulated with DNA vaccines have undergone clinical trials in humans, either as immunostimulatory sequences fused to the sequence of the target antigen or encoded by separate plasmids.

**Delivery & administration routes of DNA vaccines**

- The route of DNA vaccine administration may influence its immunogenicity.
- Intramuscular, intradermal, subcutaneous, intravenous, intranodal and intranasal administration routes have been used to elicit a desired immune response after DNA vaccination.
- There are multiple physical and chemical methods to increase the efficiency of DNA delivery into cells.
- Jet-injection, gene gun, microneedle array and electroporation are physical methods to enhance DNA vaccine delivery into cells.
- Nanoparticles can be employed for DNA vaccine delivery.

**DNA vaccines for COVID-19**

- Several preclinical and clinical trials have demonstrated the immunogenicity of DNA vaccines against SARS-CoV-2.
- preclinical and clinical trials demonstrated that DNA vaccines can elicit both humoral and cellular immune responses.
- The spike protein of SARS-CoV-2 is the main antigen used in preclinical and clinical trials of DNA vaccines for COVID-19.
- India's drug regulator has approved ZyCoV-D, the first DNA vaccine against COVID-19 for emergency use.
